# Supplementary material for: Antibiotic treatment inhibits paclitaxel chemotherapy-induced activity deficits in female mice
Source: PLoS One. 2023 May 11;18(5):e0284365. doi: 10.1371/journal.pone.0284365 (PMC10174578; doi:10.1371/journal.pone.0284365)
Supplement: S1 Fig — (DOCX) [file pone.0284365.s001.docx]

**Antibiotic treatment inhibits paclitaxel chemotherapy-induced activity deficits in female mice**

**Corena V. Grant^1,^, Kelley Jordan^1^, Melina M. Seng^1^, Leah M. Pyter*^1,2^**

^1^Institute for Behavioral Medicine Research, Ohio State University, Columbus, Ohio, USA.

^2^Department of Psychiatry and Behavioral Health, Ohio State University, Columbus, Ohio, USA

Short Title: Antibiotics and chemotherapy-induced activity deficits

*Corresponding author: Leah M. Pyter

Ohio State University

223 Institute for Behavioral Medicine Research

460 Medical Center Dr

Columbus OH 43210

t. 614.293.3496

f. 614.366.2097

leah.pyter@osumc.edu


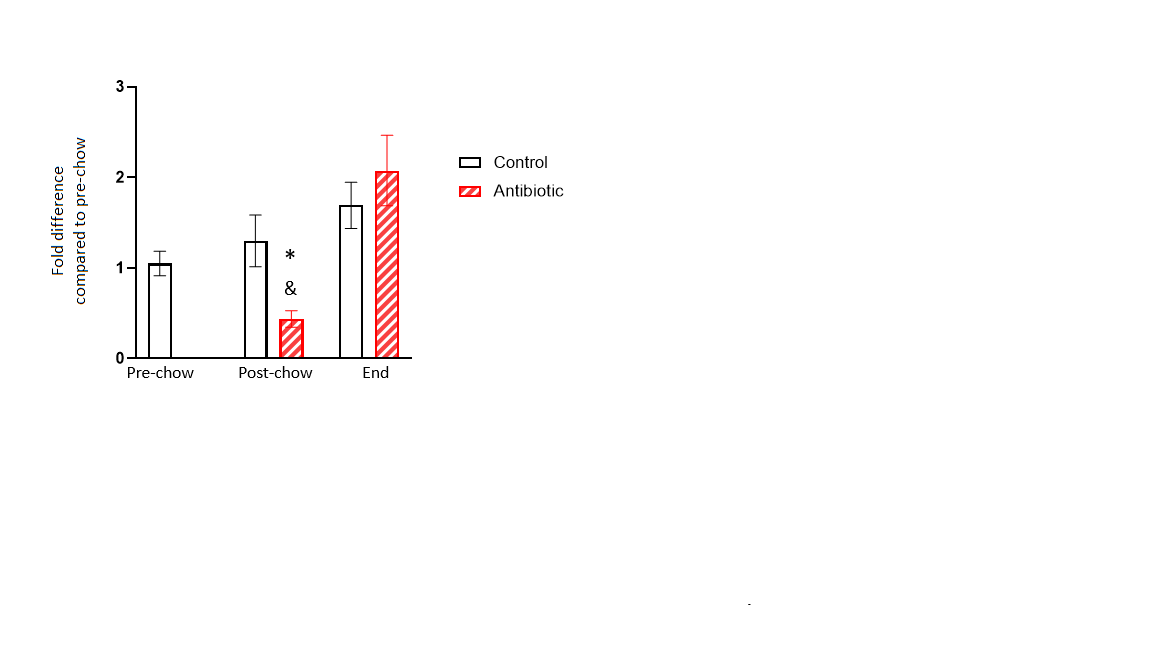


**Supplemental Figure 1. Effect of antibiotic treatment on 16s rRNA.** Fecal samples were collected from mice at 3 time points: pre-antibiotic chow, post-chow pre-vehicle treatment, and end of experiment. No fecal samples were collected from mice that received paclitaxel treatment. Pre-chow n=8, post-chow and end n=4/treatment group. Post-chow/Antibiotics vs Pre-chow *t_9_=3.139, p=0.01; Post-chow/Antibiotics vs Post-chow/Control ^&^t_6_=2.873, p=0.02
