# Supplementary material for: Antibiotic treatment inhibits paclitaxel chemotherapy-induced activity deficits in female mice
Source: PLoS One. 2023 May 11;18(5):e0284365. doi: 10.1371/journal.pone.0284365 (PMC10174578; doi:10.1371/journal.pone.0284365)
Supplement: Attachment — Submitted filename: Response to Reviewers.docx [file pone.0284365.s002.docx]

The authors would like to thank the reviewers for taking the time to critically review and provide comments on this manuscript. Each comment has been considered and revisions made to the manuscript accordingly. The authors believe that, because of these comments, the manuscript has been significantly improved. Below, please find the responses to specific reviewer comments.

Reviewers' comments:

Reviewer #1

The manuscript by Grant et al. employs antibiotics to modulate chemotherapy-induced behavioral deficits in mice, providing additional evidence that the gut microbiota likely contributes to the development of chemotherapy-associated side effects as they demonstrated in previous studies. In the current study, the authors focused on locomotor activity, weight loss/feeding behavior and endocrine responses. The study is straightforward and extends their previous work.

1. Fig 1. Was body weight during the chow/wheel running prior to chemo not monitored? This information would help support the current findings from chemo day 1 until the end of the experiment to see if weight was stable or impacted by abx prior to treatment.

The authors agree that body weight and food intake information prior to the beginning of vehicle or chemotherapy treatment would be useful for interpretation. Figure 1 has been updated as such.

The results and discussion have also been adjusted accordingly. Briefly, lines 77-90 in the results detail that during baseline, antibiotic chow consumption was initially lower than control chow; however, by the time vehicle or control treatment began, both chow groups were eating the same amount. This pattern was likewise observed for baseline body mass. However, during treatment and recovery, antibiotic chow consumption again fell below that of control chow, although body mass was only affected by chemotherapy during this period.

2. Fig 1. How was food intake measured? This is typically done in a metabolic cage, which is known to be stressful. In this case, stress would be an additional variable to consider in the context of weight loss and behavior.

For this experiment, mice were singly housed in standard cages and provided with a running wheel that acts as both a measure of activity and a form of enrichment. This allowed us to simply weigh the food and calculate how much a single animal had consumed over the time period, then average that for a g/24 h unit of measurement. Care was taken to include food pieces that were scattered on the floor of the cage in the food weight. This has been included on line 225 in the “Materials and Methods” section.

3. Figure 2. It is unclear why the authors highlight the dark phase revolutions on the day of the open field.
   a. What is the significance of this data to the overall effect?

We have highlighted in the text on lines 98 and 107 that wheel running was assessed during the dark phase because that is when mice are most active (line 98). We chose a focused visualization of wheel behavior during the dark phase on the same day/time that corresponds with the open field data (line 107). This is important because it shows congruence between fatigue behaviors as measured by running wheels and open field.

b. How is the significant decrease overall in activity caused by the OFT impacting the differences highlighted between the treatment groups? For example, it appears that a decrease in revolutions is caused by ABX in the vehicle group on open field day that was not observed in the average of the treatment days – what does this reduction in activity reflect in the abx group? Perhaps this simply reflects the choice of study design.
   The left panel of 2A is hard to read as currently presented and it would be recommended to make the x-axis longer to allow visualization of the points more clearly. Since the ABX+vehicle appear to have a decrease in activity on the chemo days despite not having chemo it would appear that some of the effect is investigator driven (initiated by the presence of the investigator treating other animals). This should be discussed.
   Panel C – units would be helpful to understand the panel easier.
   Why didn’t the authors include additional OFT parameters such as total distance traveled, or time spend in the periphery/center of arena to gain additional insight into anxiety-like behavior or total movement?

As the reviewer points out, removing mice from the wheel running cage to complete the OFT does cause a slight reduction in overall activity, however, mice are returned immediately to wheel cages after the OFT to minimize the time away. Additionally, the effect of completing OFT will have affected all groups equally.

The size of Figure 2A, left panel has been increased to improve visibility.

The authors agree, that there is an effect of receiving injections on all groups, demonstrated by the reduction in wheel revolution compared to the recovery days prior. However, the more pronounced reduction in Abx-Veh vs Control-Veh is attributed to also receiving antibiotics, which can cause some minor sickness behaviors (see food intake). This is now included in the Discussion on line 180: “Observed in both behavioral tasks, is a reduction in activity in Abx-Veh mice compared to Control-Veh mice. This would suggest that disruption of a healthy gut microbiome is detrimental and may cause fatigue behaviors.”

The y-axis has been adjusted to include units in **Fig 2C.**

The scope of this short communication focused on fatigue behaviors. We have explored anxiety-like behavior in a previous publication (Grant et al., 2021, *Brain, Behav., Immun.*). When using automatic photobeam chambers such as the ones used in this study as compared to a rodent tracking system, beam breaks are more accurate than measures of distance.

4. Why were leptin/ghrelin samples taken at different points in the L/D cycle?
   a. If animals were not fasted as is typically performed in these measurements, why were day 8 samples not taken during the light phase? Would it not be interesting to compare the impacts of these two timepoints within each group, rather than the effects across groups? This analysis was not included.

The reviewer makes a good point that the tissues are not collected at the same time point and therefore comparisons cannot be made across time points. However, the decision to collect blood from mice 1 day post chemotherapy in the late light phase (1300) was made to minimize the impact of RO eye bleed on dark phase animal behavior. However, the final tissue collection (8 days post chemotherapy) occurred in the early dark phase (1400-1800) to capture biological samples that reflect the most active point in animal behavior. Given the circadian rhythmicity of these two hormones (Kalra, et al., 2003, *Regulatory Peptides)*, the interpretation of a comparison of these two timepoints within group would be hazardous.

5. What is ClpB? Not currently defined.

The full name for ClpB (caseinolytic peptidase B protein homolog) is now included on line 58 and the abbreviation has been eliminated because this protein is only mentioned once in the manuscript.

6. Please avoid use of “tended” to describe changes. If they are not significant, they are not different. If they are “trending” then be sure to highlight the rationale for pointing out a non-significant effect, including low n number or high variability.

Tended was used in 2 instances in this manuscript and it has been removed in both locations. First, it was used to describe the data that led to a Chow x Treatment effect in wheel running when there were no significant comparisons using a Dunnett’s Multiple Comparisons test completed after the 2-way repeated measures ANOVA. This portion on line 101 now reads “Antibiotics interacted with chemotherapy treatment (Fig 2A, Chow x Treatment effect, F1,25=5.4, p<0.05), driven by decreased activity in antibiotic versus control chow-fed mice in within the vehicle treatment group, but increased activity in antibiotic versus control chow-fed mice in within the chemotherapy treatment group.”

The second location is in reference to a statistical analysis resulting in a p=0.06 value, and it has been adjusted in line 124 as follows: “One day after treatment completion, a trend for chemotherapy to increase ghrelin was observed; this likely did not reach significance because of high variability in the Control-Chemo group (Fig 3A, Treatment effect F1,21=4.0, p=0.06).”

7. “Chemotherapy and ABX energy-regulating systems” needs to be clarified. Modulate perhaps is what the authors meant?

We agree this needed some clarification, the title of this section has been adjusted on line 122 to “Chemotherapy and antibiotics modulate energy-regulating systems”

Reviewer #2:

8. Author claims that antibiotic cocktail treatment alters the gut microbiota in chemotherapy treated mice which in turn attenuates the fatigue like behavior by modulating the melanocortin axis. The major concern is that author has not shown any microbial consortium in these mice model. Authors should show the microbial diversity in every conditions.

While microbial diversity is not reported in these specific animals, this exact antibiotic treatment regimen was used in a previous publication of ours (Grant et al., 2021). That publication reports an overall reduction in alpha diversity and reductions in the relative abundance of approximately 100 genera. This is included in the Methods section of the manuscript on line 243.

9. Chemotherapy and antibiotics (Abx-chemo) both treatments cause the decline in body weight. Is there any significant difference in between these two treatments? In both the cases it starts to recover after 16 and become almost similar on later time points. What is the most probable reason for this enhancement?

During the treatment period, there is only a decline in body mass associated with chemotherapy treatment, which occurs regardless of chow treatment. The body mass recovery in the Control-Chemo and Abx-Chemo groups is likely a result of cessation of chemotherapy treatment. To address this comment and comment 1 by Reviewer 1, the results section on body mass has been expanded on lines 77-90.

10. In methodology section, author mentioned the treatment groups consisted of Control-Vehicle, Antibiotics-Vehicle (Abx-Vehicle), Control-Chemotherapy (Control-Chemo), and Antibiotics-Vehicle (Abx-Chemo). What is the difference in between the Antibiotics-Vehicle (Abx-Vehicle) and Antibiotics-Vehicle (Abx-Chemo). If both are similar why two different symbols have been used.

We appreciate the reviewer catching this mistake in nomenclature. This is a 2x2 study design and “Vehicle” has been changed to “Chemotherapy” as such on line 233.

11. In fig.2, wheel running activity also showing similarity in control-chemo and Abx-chemo group. It begins to increase and was almost similar in all groups on day 19. Why such changes have been happened.

Yes, there is definitely a similarity between Control-Chemo and Abx-Chemo groups because of the strong fatigue behavior induced by chemotherapy treatment. However, as highlighted in Fig 2A middle and right panel, there is only a significant difference between vehicle and chemotherapy treatment in the control chow group, not the antibiotic chow group, demonstrating that antibiotics modulates this chemotherapy effect. The similar wheel running on day 19 is likely driven by a recovery from treatment (chemotherapy treated groups) and the absence of stress associated with injections (vehicle group). This has been added to the Discussion on line 183 with the sentence “It is also notable that, by the completion of this experiment, all animals had roughly the same running wheel activity thereby suggesting that chemotherapy-associated fatigue recovers over time.”

12. Is there any mortality in this experiment?

Two animals were euthanized because they lost greater than 20% of their body mass with chemotherapy treatment. This is stated in the Methods on line 252.

13. It will be good if author would measure the level of POMC protein level in serum.

This is a great suggestion, unfortunately, after running the Leptin/Ghrelin assay, there is not enough blood left to complete this.
