# Supplementary material for: Antibiotic treatment inhibits paclitaxel chemotherapy-induced activity deficits in female mice
Source: PLoS One. 2023 May 11;18(5):e0284365. doi: 10.1371/journal.pone.0284365 (PMC10174578; doi:10.1371/journal.pone.0284365)
Supplement: Attachment — Submitted filename: Response to reviewers round 2b.docx [file pone.0284365.s003.docx]

In addition to the original revisions that we previously submitted in response to Reviewers 1 and 2, we appreciate the time and effort Reviewer #3 has taken to review our manuscript. Please see our detailed responses below for how comments were incorporated into the manuscript. Reviewers 1 and 2 comments were deemed as adequately addressed in the first round of revisions.

1. Throughout the manuscript the authors need to clearly state which chemotherapy regimen was used in the study. Presently, the exact chemotherapy is only stated in the title of the paper and infrequently in the methods. It is well established that different types of chemotherapy induce specific side effects. The potential effects on different chemotherapy regimens must be discussed.

Throughout the manuscript, “chemotherapy” was replaced with “paclitaxel,” the specific chemotherapeutic agent used in this study.

In the Conclusions, the following is now included beginning on line 226.

“Given the modest sample size, future studies should replicate these findings, further investigate the effect of antibiotics on reward motivation and effort aspects of fatigue, and study fatigue caused by other types of commonly used chemotherapeutics.”

2. Regardless of prior reports, verification that the gut microbiome was altered by the antibiotic treatment is needed. Were any bacteria remaining? One antibiotic treatment was terminated, how fast did bacteria return and which bacteria returned? Is there a difference if the antibiotic treatment was terminated at the same time as the chemotherapy?

We have previously published 16S sequencing of bacterial community DNA using this antibiotic and chemotherapy paradigm (Grant et al., 2021). In the Methods, we added a statement with the reference to this previous comprehensive gut bacterial analysis as follows: “This treatment was previously demonstrated to significantly decrease gut bacterial alpha diversity and reduce the relative abundance of about 100 genera in the colon.[15]”

Fecal samples from the present study were not collected from all mice at all points of the experiment and budget restrictions prevented re-sequencing, however, we were able to complete quantification of the total fecal bacterial 16S gene from a subset of these mice. Consistent with our previous work, the graph here demonstrates that mice that received antibiotic chow had a lower amount of gut bacteria then mice that received control chow (*p=0.03) and compared to pre-chow gut bacteria content (& p=0.01). The recovery of overall bacterial levels at the end of the experiment is likely the result of antibiotic-resistant bacterial taxa blooming in the gut as this analysis represents total bacteria, irrespective of relative abundances of specific taxa. Indeed antibiotics reduce diversity of the gut microbial community without wiping out all types of bacteria; some thrive when others are depleted (Lange et al., 2016).

3. The decreased food intake with antibiotic treatment is a confounding factor in these studies. More controls for food intake are needed. In Figure 1 the body mass decreased with chemotherapy with and without antibiotics, as would be expected. But the food intake (panel C) does not match this difference. During treatment, both antibiotic treated groups have decreased food intake. More discussion about this discrepancy is needed.

This study was designed as a standard 2 x 2 experimental design with a total of 4 groups +/- Antibiotic chow and +/- Paclitaxel chemotherapy, therefore we believe this study is properly controlled. The authors agree that the dichotomy between food intake and body mass is puzzling, however, chemotherapy and antibiotic treatment affects metabolism thereby changing the expectation that an increase in food intake leads to an increase in weight.

This is thoroughly discussed for readers beginning on line 154 in the Discussion. [The underlined section was added to the original discussion].

“As previously published, this paclitaxel chemotherapy regimen transiently reduced body mass compared to vehicle-treated mice,[18] which was not due to a difference in food intake. However, antibiotic-treated mice ate significantly less chow than control mice without an impact on body mass. It is probable that this is due to the aversive taste of antibiotics because the reduction is observed throughout the entire experiment, and others have shown that mice reduce water intake when the water is formulated with a similar antibiotic cocktail containing ampicillin, vancomycin, neomycin, and metronidazole.[19] Interestingly, during baseline acclimation of food and running wheels, mice initially consumed less antibiotic-containing food, however by the time of vehicle or paclitaxel treatment, that was not the case. During the course of treatment, though, mice again consumed less antibiotic-containing chow, however weight loss was only impacted by chemotherapy treatment, not a decrease in antibiotic food intake. This is likely due to the metabolic changes documented in patients treated with chemotherapy.[20] Further, the increase in ghrelin and decrease in leptin caused by antibiotics (measured 8 days post treatment) would suggest food intake decreases are occurring despite appetite-stimulating signals. Despite the reduced food intake, we have previously demonstrated that this antibiotic chow is effective at decreasing gut bacterial alpha diversity.[15]”

4. Figure 2A shows no effect of the antibiotic treatment on the number of revolutions (compare values shown as open and closed red curves). And the right bar graph of panel 2A shows no improvement with the addition of antibiotic treatment in the paclitaxel treated mice. Protection with antibiotic treatment may be present in the open field test, but the number of mice included is small and the effect is quite modest. Is the difference between the two groups - chemotherapy with and without antibiotics – significantly different? Regardless, more mice need to be tested before a conclusion can be asserted because the variance is very large.

As indicated in Figure 2, there is not a significant difference between Control-Chemo and Abx-Chemo in either wheel running or open field. However, this specific post-hoc analysis type of question is not the main goal of the study. The goal of this study was to identify the main effects and interactions of Antibiotics and Chemotherapy treatment. Unfortunately, the addition of mice to the study at this time is beyond our budget. Replication of these findings in future studies is now listed in the Conclusions beginning on line 226.

“Given the modest sample size, future studies should replicate these findings, further investigate the effect of antibiotics on reward motivation and effort aspects of fatigue, and study fatigue caused by other types of commonly used chemotherapeutics.”

5. In Figure 3, the main effects on ghrelin and leptin (panels A and B) appears to be changes with antibiotic treatment, regardless of the presence of paclitaxel treatment. At one day post treatment there may be modest differences, but at 8 days post treatment, no differences are presented. Panels C-G also only show antibiotic effects, but no difference with and without chemotherapy.

This is correct, the main effects on *Pomc* and *Htr2c* expression are from antibiotic treatment. Regardless of the lack of chemotherapy effect, we found it important to include these results so that we could provide a complete discussion of alteration of metabolic pathways.

6. Need to compare the results presented here with previously published results showing that paclitaxel treatment did not alter locomotion (for example, Nguyen et al, Molecular Neurodegeneration, 2021).

We have included the suggested reference and one other that did not demonstrate a reduction in locomotor activity with paclitaxel treatment, however, we are careful to point out that the dose of paclitaxel used in these references are significantly lower that the 30 mg/kg dose that we use in this manuscript. The changes are incorporated on beginning on line 174.

“This is also consistent with the data of others demonstrating a reduction in dark phase horizontal locomotor activity and wheel running.[21] However, some groups have not observed a reduction in locomotion in mice treated with paclitaxel, [22,23] likely due to differences between our high-dose of paclitaxel and treatment with a much lower dose (2 or 10 mg/kg).”

7. The present study only examined the effects that persisted 8 days after completion of treatment. Many side effects of chemotherapy persist for prolonged periods. The authors need to include experiments that address the long duration of the side effects. Does the proposed protection persist after terminating chemotherapy only when antibiotic treatment is maintained or does protection persist when both chemotherapy and antibiotic treatment terminate at the same time?

The authors agree that there is a need in this field to identify the temporal effects of antibiotic treatment on chemotherapy-associated fatigue. However, these studies were beyond the scope and budget of the current study.

8. Does the proposed protection alter the ability to treat tumor size and/or spread to additional tissues?

The impact of antibiotic treatment on the anti-cancer effects of paclitaxel are not explored here. Prior to moving to moving towards a more clinically-relevant study, it would be essential to determine if there was an interaction between paclitaxel chemotherapy and antibiotic treatment that prevented effective cancer treatment.

9. In the statistical measurements shown on the figures, it is not clear which bars are being compared. Figures should be easily understood, without the need to read the entire text. Please include clarification on the figures.

The authors appreciate Reviewer 3 catching this, the meaning of * and # has been added to Figure legends 2 and 3.
