# Supplementary material for: Antibiotic treatment inhibits paclitaxel chemotherapy-induced activity deficits in female mice
Source: PLoS One. 2023 May 11;18(5):e0284365. doi: 10.1371/journal.pone.0284365 (PMC10174578; doi:10.1371/journal.pone.0284365)
Supplement: Attachment — Submitted filename: Response to Reviewers.docx [file pone.0284365.s004.docx]

The authors would like to thank the Editor and Reviewer for a final chance to make revisions to this manuscript. Importantly, we have now included a full paragraph of limitations and carefully incorporated changes based on the other comments, as detailed below.

Reviewer Comments

1) It is noted that chemotherapy regimen is now clearly stated throughout. Note also that the marked copy of the manuscript does not match the revision at the beginning of the file.

We agree that it is difficult to go back to the original submission and create a marked copy from all 3 rounds of revisions. However, for the marked version here we have included those of revision 2 in red and this third revision in blue and purple. The clean copy was created as the last step of the resubmission process to ensure no changes were made that were not on the marked copy.

2) There must be a section devoted to limitations. This section needs to include:
Only female mice were included in the study.
The number of mice included in each group is very limited.
The measured effects on were small and had a large variance. This includes the effects on ghrelin and leptin. The effect on activity shows a trend, but was not significant.
The effect of antibiotic treatment on the ability to shrink tumors was not assessed.
The effects of antibiotic treatment was not specific for chemotherapy, that the antibiotic treatment altered the gut microbiome regardless of the presence of paclitaxel.
Bacterial levels return by the end of antibiotic treatment. This response may impact other aspects of cancer treatment.
The present study only examined the effects that persisted 8 days after completion of treatment. Further studies are needed because many side effects of chemotherapy persist for prolonged periods (month to years).

In response to this comment, we have added a paragraph in the Discussion dedicated to the limitations listed above. All limitations listed above were addressed and the paragraph beginning on line 231 reads as follows:

The results of this study are modest and therefore should be regarded in the context of other bodies of work. Future studies will need to address these limitations to significantly impact the understanding of how the gut microbiome plays a role in the development of chemotherapy-associated fatigue. This study was conducted only in female mice and with relatively small sample sizes (n=6-8/group), therefore the statistical power was limited and variability of the biology and activity results were significant. While paclitaxel chemotherapy is used in the treatment of cancer, mice in this study were tumor-naive. Including tumor-bearing mice would provide a more complete understanding of fatigue in cancer patients. The antibiotic treatment used in this study targeted both gram-positive and -negative bacteria and was administered for the entirety of the study to modify the gut microbiome. Future manipulation of the antibiotics regimen and schedule could address the potential for antibiotics, used periodically to resolve infection in chemotherapy patients, to modulate chemotherapy-associated fatigue and energy homeostasis. Furthermore, bacterial sequencing was not repeated for these mice as in our previous study which demonstrated both antibiotic and chemotherapy consequences for the gut relative bacterial taxa abundances.[15] Future bacterial sequencing would allow for correlation analyses between locomotion, hypothalamic PCR, hormone plasma concentrations, and specific gut taxa. Lastly, chemotherapy-associated side effects are known to be persistent in some patients, therefore the focus of this study acutely after treatment does not capture the potential effects of antibiotics on long-term side effects of paclitaxel.

3) As stated previously: regardless of prior reports, verification that the gut microbiome was altered by the antibiotic treatment must be included. The figure included in the response to reviewers needs to be included in the manuscript. This statement needs to be included: “The recovery of overall bacterial levels at the end of the experiment is likely the result of antibiotic-resistant bacterial taxa blooming in the gut as this analysis represents total bacteria, irrespective of relative abundances of specific taxa. Indeed antibiotics reduce diversity of the gut microbial community without wiping out all types of bacteria; some thrive when others are depleted (Lange et al., 2016).”

The graph that was original in the response to reviewers is now included as a supplemental figure. We have included the detail of this result on line 79 and line 91 which now read as follows:

(line 79) As expected, this antibiotic cocktail in chow caused a significant decrease in fecal 16S rRNA as verified in vehicle-treated mice (Supplemental Fig1).

(line 91) By the end of the paclitaxel recovery period, total fecal bacterial DNA (16S rRNA) returned to pre-antibiotic levels despite continuous antibiotic treatment (Supplemental Fig1).

In the discussion we have included this on line 174 which now reads “Further demonstrated here is a recovery of the levels of fecal bacterial DNA (16S rRNA) by the end of the experiment despite continuous antibiotic treatment. This is consistent with the current knowledge that following an initial reduction in bacterial load, resilient bacterial communities will repopulate the gut community.[20]”

4) As previously stated the authors need to compare the results presented here with previously published results showing that paclitaxel treatment did not alter locomotion (for example, Nguyen et al, Molecular Neurodegeneration, 2021). The authors state:
   “We have included the suggested reference and one other that did not demonstrate a reduction in locomotor activity with paclitaxel treatment, however, we are careful to point out that the dose of paclitaxel used in these references are significantly lower that the 30 mg/kg dose that we use in this manuscript. The changes are incorporated on beginning on line 174.”
   The citations included are not obviously related to the topic (refs 21,22, 23 appear to relate to statistical analysis and the microbiota).

We appreciate the that the reviewer identified this error with references. There was a mix up in the order of references and a couple seemed to be missing. The references have been corrected to their proper order and now include the references intended to address this comment in the prior resubmission.

21.	Ray MA, Trammell RA, Verhulst S, Ran S, Toth LA. Development of a mouse model for assessing fatigue during chemotherapy. 2011; 61:2

22.	Toma W, Kyte SL, Bagdas D, Alkhlaif Y, Alsharari SD, Lichtman AH, et al. Effects of paclitaxel on the development of neuropathy and affective behaviors in the mouse. Neuropharmacology. 2017;117: 305–15. doi:10.1016/J.NEUROPHARM.2017.02.020

23.	Nguyen LD, Fischer TT, and Erlich BE. Pharmacological rescue of cognitive function in a mouse model of chemobrain. Mol Neurodegener. 2021;16:41. doi.org/10.1186/s13024-021-00463-2

5) Also, the statement that the treatment levels in the current manuscript are much higher than previous work is not a correct statement. Paclitaxel treatment is often at least 20 mg/kg in previous studies.

We have removed the qualifier “much” and instead reported the cumulative dose of our treatment regimen, to capture both dose and frequency, for direct comparison to the cumulative dose in the papers we reference for more detail in how the dosing difference could cause locomotion discrepancies between studies.

Line 185 now reads as follows: However, some groups have not observed a reduction in locomotion in mice treated with paclitaxel, [22,23] likely due to differences between our higher-dose paclitaxel (cumulative 180 mg/kg) and those with a lower dose of paclitaxel (cumulative 4 or 80 mg/kg, respectively).

6) As for the figures, it is still not clear which data bars are being compared. There are brackets over all bars in many panel, but it is not clear which comparison is related to the p value presented. Figures need to be updated.

We apologize for the confusion. The p-values listed above bar graphs are the ANOVA main effect results, therefore they do not relate to any pairwise comparison. Indication that these are main effect p-values has been added in the figure legends. This is also the case for brackets above the two portions of graphs in figure 1. Again, it is now indicated in the figure legend that these p-values are those of the ANOVA main effects.

Figure legend 1, line 98 now reads: The average food intake per mouse per 24-hour period. Each point reflects the previous 48 hr. n=5-8. p-values are representative of ANOVA main effects over the indicated time period.

Figure legend 2 and 3 now both include the statement “p-values above brackets are ANOVA main effect results”

7) There is a new author, but this author has no contribution listed on lines 322-324

We thank the Reviewer for catching this detail. The new authors contributions have been added.

Line 352 now reads as follows: Conceptualization (L.P., K.J.), data curation (K.J., M.S.), data analysis (K.J., C.G., M.S.), funding acquisition (L.P.), manuscript preparation (C.G.). All authors reviewed, edited, and approved the final draft of the manuscript.

Editor Comments

1) Many of the arguments made in the response to reviewers regarding the limitations of the study need to be included in the discussion.

As indicated in Reviewer comment #2, there is now a complete limitations paragraph included in the discussion to more completely address concerns including sample size and bacterial sequencing.

2) Since the number of samples is insufficient to adequately address some of the conclusions, the authors need to very clearly note that this is a pilot study up front and need to clearly outline the specific limitations, as pointed out by Reviewer 3. Simply stating that there was insufficient budget is not an adequate response for lack of experimental rigor.

It is now made more clear in the Conclusions that this study needs to be followed up with future work. Line 254 now reads “Given the modest sample size (n=6-8), this work may be regarded as preliminary data for future studies. Replication of these findings, further investigation of the effects of antibiotics on reward motivation and effort aspects of fatigue, and studying fatigue caused by other types of commonly used chemotherapeutics will all be important questions for future work.”

See response to Reviewer comment #2 for how we addressed limitations.
